# Fire Smoke Elevated the Carbonaceous PM_2.5_ Concentration and Mortality Burden in the Contiguous U.S. and Southern Canada

**DOI:** 10.21203/rs.3.rs-5478994/v1

**Published:** 2024-11-21

**Authors:** Zhihao Jin, Gonzalo A. Ferrada, Danlu Zhang, Noah Scovronick, Joshua S. Fu, Kai Chen, Yang Liu

**Affiliations:** Gangarosa Department of Environmental Health, Rollins School of Public Health, Emory University; NOAA Earth System Research Laboratories; Deparent of Biostatistics, Rollins School of Public Health, Emory University; Gangarosa Department of Environmental Health, Rollins School of Public Health, Emory University; Deparent of Civil and Environmental Engineering, University of Tennessee; Department of Environmental Health Sciences, Yale School of Public Health; Gangarosa Department of Environmental Health, Rollins School of Public Health, Emory University

**Keywords:** fire smoke PM2.5, mortality, carbonaceous PM2.5, wildland fire, prescribed fire

## Abstract

Despite emerging evidence on the health impacts of fine particulate matter (PM_2.5_) from wildland fire smoke, the specific effects of PM_2.5_ composition on health outcomes remain uncertain. We developed a three-level, chemical transport model-based framework to estimate daily full-coverage concentrations of smoke-derived carbonaceous PM_2.5_, specifically Organic Carbon (OC) and Elemental Carbon (EC), at a 1 km^2^ spatial resolution from 2002 to 2019 across the contiguous U.S. (CONUS) and Southern Canada (SC). Cross-validation demonstrated that the framework performed well at both the daily and monthly levels. Modeling results indicated that increases in wildland fire smoke have offset approximately one-third of the improvements in background air quality. In recent years, wildland fire smoke has become more frequent and carbonaceous PM_2.5_ concentrations have intensified, especially in the Western CONUS and Southwestern Canada. Smoke exposure is also occurring earlier throughout the year, leading to more population being exposed. We estimated that long-term exposure to fire smoke carbonaceous PM_2.5_ is responsible for 7,462 and 259 non-accidental deaths annually in the CONUS and SC, respectively, with associated annual monetized damage of 68.4 billion USD for the CONUS and 1.97 billion CAD for SC. The Southeastern CONUS, where prescribed fires are prevalent, contributed most to these health impacts and monetized damages. Given the challenges posed by climate change for managing prescribed and wildland fires, our findings offer critical insights to inform policy development and assess future health burdens associated with fire smoke exposure.

## Introduction

Over the past half-century, wildland fire activity has significantly increased, not only in the U.S. but also in other temperate and high-latitude ecosystems, including Canada and Europe (1, 2). Notably, human-induced climate change was responsible for an additional 4.2 million hectares of forest fire area between 1984 and 2015, nearly doubling the area expected to be affected by fire in the absence of climate change (3). As a result of climate change, large-scale wildland fire events have become more frequent and intense, and fire seasons have lengthened in the Contiguous U.S. (CONUS) in recent decades. Previous research has indicated that wildland fire smoke have accounted for nearly 25% of the ambient fine particulate matter (PM_2.5_, particles with a diameter of less than 2.5 μm) across the U.S. in recent years, and up to 50% in certain Western U.S. regions (4).

One of the primary wildfire management strategies is prescribed burning. Prescribed fires not only reduce the biomass available for subsequent wildfires, but they also support carbon sequestration, facilitate ecological resilience, and play a critical role in restoring fire-adapted ecosystems that have been degraded due to decades of fire exclusion (5, 6). Over 65% of the prescribed burn areas are in the Southeastern U.S (7). This imbalance in the application of prescribed fires has resulted in comparable regional average PM emissions from prescribed fires in the Southeastern U.S. and wildfires in the Western U.S. (8). In the context of climate change, as the use of prescribed burning is increasing to control wildfires, smoke from these burns is becoming a growing public health concern, particularly in the Southeastern U.S. (9, 10). The National Prescribed Fire Acts (116th and 118th Congress) emphasizes the importance of public health and safety risks associated with the expanded use of prescribed fires. However, it states that smoke from prescribed fires is generally less harmful and of shorter duration compared to wildfire smoke, stating that it exposes children to fewer adverse health effects. Such a statement, however, is based on limited research, which may lead to an underestimation of prescribed burning’s health risks.

With anthropogenic climate change playing an increasingly critical role in escalating wildfire activity, the negative impacts of smoke on air quality and public health are likely to worsen in the future (11). Fire smoke contains considerable amount of PM_2.5_, significantly deteriorating the air quality in downwind communities that are tens to hundreds of kilometers away (12). Smoke PM_2.5_ is characterized by substantial concentrations of carbonaceous matter, including organic carbon (OC) and elemental carbon (EC), which are produced by the combustion and incomplete burning of organic materials such as wood, leaves, and other vegetation. This distinguishes fire smoke PM_2.5_ from typical ambient PM_2.5_, which tends to present greater oxidative potential (13–15). The unique characteristics of fire smoke PM_2.5_ may alter the composition of regional PM_2.5_ and potentially affect its toxicity.

While numerous studies have linked exposure to PM_2.5_ with various adverse health impacts (16–19), epidemiological research linking exposure to fire smoke PM_2.5_ with adverse health outcomes is still in its early stage. Long-term exposure to smoke PM_2.5_ has been linked to all-cause mortality in the CONUS, particularly among vulnerable populations such as the elderly (20). It is estimated that 11,415 non-accidental deaths per year in the CONUS can be attributed to smoke PM_2.5_, with cardiovascular diseases contributing the most (20). Short-term exposure to wildfire smoke PM_2.5_ has been associated with increased risks of respiratory morbidity, mental health issues, and excess mortality, (21–24). Evidence on the health effects of different chemical components of smoke PM_2.5_ is sparse. For example, OC has been identified to be an important component influencing PM_2.5_ toxicity to several reactions harming organic systems and a key contributor to all-cause mortality (25–27). EC, due to its small size, can penetrate deeply into the respiratory tract and serve as a transporter for various toxic substances (28).

Research on the health effects of smoke PM_2.5_ has been hindered owing to the scarcity of long-term exposure data, especially data with comprehensive spatial coverage and high spatial-temporal resolution. Most epidemiological studies on smoke PM have relied on local ground-based monitoring stations, satellite images, uncalibrated chemical transport model simulations (CTM) or simple classifications of smoke-affected areas to investigate the health impacts of fire smoke (29–34). These methods were either based on coarse resolution smoke estimates or did not quantify smoke PM, potentially introducing exposure misclassification. Emerging research has shown great promise to generate long-term and high-resolution smoke-derived PM_2.5_ concentrations by calibrating CTM simulations. For instance, Cleland et al. tested the model performance of predicting 1 km^2^ wildfire smoke PM_2.5_ based on CTMs simulations and different combinations of concentration datasets (35).

The model that fused ground-based observations, satellite aerosol optical depth (AOD)-derived concentrations and CTMs simulations provided the best estimate (R^2^ = 0.71) in fire-impacted regions, highlighting the importance of integrating multiple datasets. Similarly, Zhang et al. developed CMAQ-based models to estimate daily 1 km^2^ smoke PM_2.5_ total mass, which achieved strong model performance with R^2^ of 0.75 and 0.68 in smoke-impacted regions and non-smoke regions, respectively (36). Nevertheless, few studies have adopted CTM-based models to estimate smoke PM_2.5_ speciation with high spatial and temporal resolution. This is largely because CTM simulations for PM_2.5_ speciation often face higher uncertainties compared to those for total PM_2.5_ mass, demanding more advanced calibration techniques (37, 38).

In this study, we developed a three-level, CTM-based modeling framework to estimate daily concentrations of fire smoke carbonaceous PM_2.5_, specifically OC and EC, at 1 km^2^ spatial resolution from 2002 to 2019 with full coverage across the CONUS and Southern Canada (SC). This framework integrated information from CMAQ simulations of PM_2.5_ mass and speciation, ground-based observations and multiple auxiliary spatial and spatiotemporal datasets. This innovative approach allows us to fill important research gaps described above, namely, to differentiate exposure by specific carbonaceous constituents of smoke PM_2.5_, and to estimate fire smoke-related health burdens over the long-term. By leveraging the high spatial and temporal resolution of our model predictions, we then analyzed the spatiotemporal patterns of smoke impact frequency and concentrations of carbonaceous PM_2.5_ from fires smoke. Furthermore, we estimated the populations exposed to fire smoke. Lastly, we investigated the impacts of long-term exposure to fire smoke on mortality burden and associated monetized damages.

## Results

### Model performance

The CV results revealed strong model performance (Table S1-S6). For daily-level predictions, Smoke-off models showed higher accuracy, with random CV R^2^ values above 0.75 for base learners and 0.77 for meta-learners. Meta-learners for EC achieved average random CV R^2^ values of 0.80 in Smoke-off and 0.71 in Smoke-on scenarios, while OC performance dropped in Smoke-on with a reduced R^2^ of 0.67 and increased RMSE of 1.20 μg/m^3^. Spatial CV showed low prediction errors for OC and EC in Smoke-off (Fig. S1), with RMSEs below 0.8 μg/m^3^ and 0.2 μg/m^3^, respectively. In contrast, Smoke-on scenarios exhibited relatively higher RMSEs, especially in areas prone to fire smoke. For temporal CV, R^2^ values were consistently higher for Smoke-off scenarios (Fig. S2), ranging between 0.65 and 0.85. In Smoke-on scenarios, OC and EC displayed fluctuate R^2^ values between 0.58 and 0.65, with notable declines in 2002. At monthly and annual levels, model performance improved significantly (Table S1–S6). Adjustments to residuals through GAMs further enhanced accuracy (Table S7), with random CV R^2^ values surpassing 0.91 for monthly and 0.97 for annual predictions. Most overestimations and underestimations observed at the daily level were reduced by averaging at the monthly level (Fig. S3). Overall, the RASL model demonstrates reliable daily predictions across various CV experiments and further improvements in adjusting the long-term prediction residuals. Feature importance analysis (Fig. S4) indicated CMAQ simulations of carbonaceous PM_2.5_ were primary predictors, with MAIAC AOD, urbanization factors, meteorological factors, and spatial and temporal characteristics also influential.

### Spatial and temporal patterns of smoke carbonaceous PM

Figure S5 illustrates the average number of smoke days per year across our study domain. The central-south (i.e., Missouri, Arkansas, and Oklahoma) and southeastern (Alabama, Georgia, and Florida) regions of the CONUS exhibited the highest average number of smoke days (200 + days per year). The Western CONUS (i.e., California, Oregon, Idaho, and Montana), North-Central CONUS (i.e., North Dakota, South Dakota, and Minnesota) and adjacent areas in SC such as British Columbia also experienced a significant number of smoke days (~ 150 days), though the sources of smoke in these regions differ. In the Western CONUS and SC, the primary source of smoke is wildfires. Conversely, in the central-south and southeastern regions of the CONUS, prescribed fires are the main source of smoke (39). The northeastern regions of the study domain are less frequently impacted by smoke (~ 100 days) but can still experience significant smoke pollution from long-range transport (40, 41).

**Figure 1:**
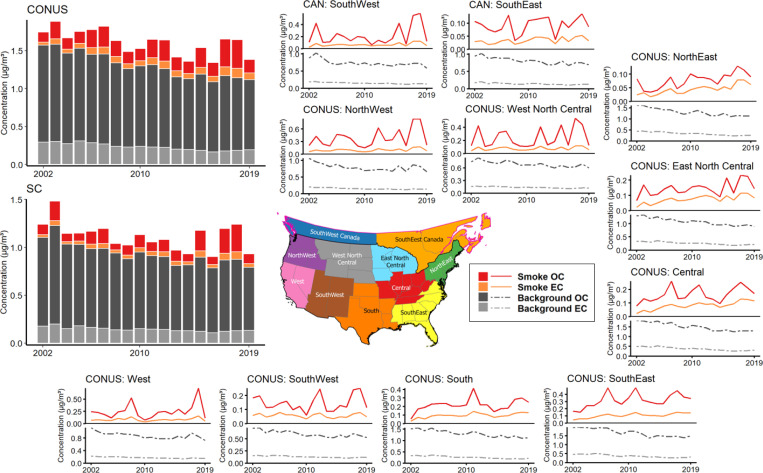
Annual average concentration of background and smoke PM_2.5_ OC and EC in the CONUS and SC, categorized by climate regions and spanning the study period from 2002 to 2019.

We summarized the background, total, and smoke-specific concentrations of PM_2.5_ OC and EC across different time scales and climate regions in Fig. S6. From 2002 to 2019, both the CONUS and SC experienced a declining trend in background carbonaceous PM_2.5_, with annual concentrations falling below 0.95 μg/m^3^ for OC and 0.20 μg/m^3^ for EC by 2019. When comparing the background carbonaceous PM_2.5_ between the periods 2002–2010 and 2011–2019, improvements in annual background PM_2.5_ were observed, with reductions of 0.27 μg/m^3^ for the CONUS and 0.17 μg/m^3^ for SC after 2011. The CONUS climate regions of SouthEast, South, Central, and NorthEast exhibited higher background concentrations and more significant reductions over the years (Fig. 1). Additionally, urban areas displayed higher background concentrations of carbonaceous PM_2.5_ (Fig. S7), with long-term average concentrations reaching approximately 2 μg/m^3^ for OC and 1 μg/m^3^ for EC. Elevated background PM_2.5_ OC levels were also common in many rural and forested areas of the South, Central and SouthEast CONUS climate regions, while high levels of PM_2.5_ EC were mostly concentrated in urban centers across the study domain.

Considering the impacts of fire smoke, we observed a distinct contribution of smoke PM_2.5_ OC, whereas the contribution of smoke PM_2.5_ EC was smaller and showed less fluctuation over the years. The mean annual concentration of smoke PM_2.5_ OC was 0.22 μg/m^3^ for the CONUS and 0.14 μg/m^3^ for SC, while smoke EC concentrations were 0.08 μg/m^3^ for the CONUS and 0.05 μg/m^3^ for SC. Combining smoke PM_2.5_ OC and EC, smoke carbonaceous PM_2.5_ accounted for 19% and 16% of the total concentrations in the CONUS and SC, respectively. At the monthly level, smoke carbonaceous PM_2.5_ exhibited a notable increase during the peak months of wildfire season (July–November) (Fig. S8), with average monthly concentrations rising to 2.38 μg/m^3^ for the CONUS and 2.60 μg/m^3^ for SC, accounting for 58% and 57% of total concentrations, respectively.

The Western and Southern CONUS climate regions (i.e., NorthWest, West, West North Central, South, and SouthEast) and the Southwestern Canada experienced more fire smoke impacts, including wildland fire and prescribed fire, resulting in increased smoke carbonaceous PM_2.5_ at both regional and national scales (Fig. 1). Additionally, the annual mean concentrations of smoke carbonaceous PM_2.5_ increased by 0.08 μg/m^3^ for the CONUS and 0.05 μg/m^3^ for SC before and after 2011. This intensifying trend in fire smoke has offset nearly one-third of the improvements in background concentration. Megafire years of 2012, 2015, 2017, and 2018 experienced significantly higher concentrations of both smoke PM_2.5_ OC and EC at the monthly and annual levels.

**Figure 2:**
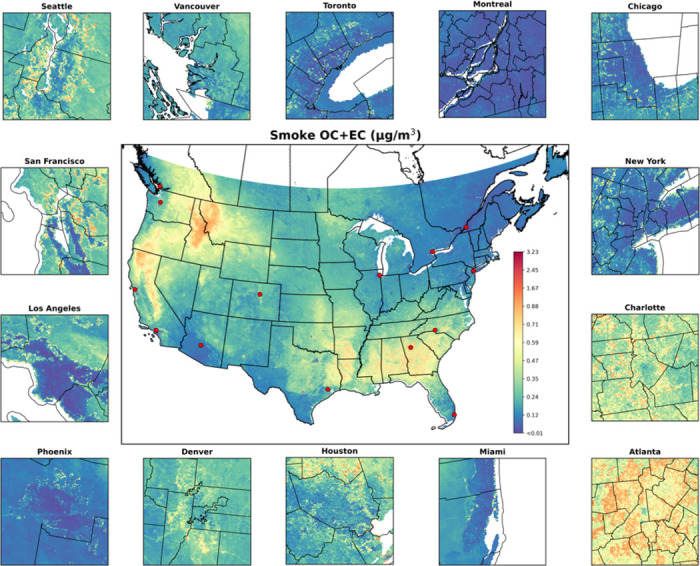
Long-term average annual concentration of smoke carbonaceous PM_2.5_ from 2002 to 2019, with major urban centers (located at red points) zoomed in for detailed visualization.

Our 1 km^2^ prediction maps revealed different spatial distributions between long-term smoke OC and EC concentrations (Fig. S7). High smoke OC concentrations (> 0.50 μg/m^3^) were primarily observed in rural areas of the Western CONUS and SC, and were sporadically distributed in the Southeastern CONUS. Elevated EC concentrations (> 0.15 μg/m^3^) were often collocated with high smoke OC, with the Southeastern CONUS, particularly Georgia, Florida, Mississippi, and Texas, exhibiting even higher smoke EC concentrations (> 0.20 μg/m^3^). Long-term average concentrations of smoke carbonaceous PM_2.5_ were highest in the Western CONUS and Southwestern Canada, particularly in California, Idaho, and Montana (Fig. 2). The Southeastern CONUS also experienced comparable concentrations of smoke carbonaceous PM_2.5_, with urban centers such as Charlotte and Atlanta, and their surrounding areas, showing higher exposure levels than urban areas in other parts of the study domain. During megafire years (2012, 2015, 2017, and 2018), elevated smoke carbonaceous PM_2.5_ were concentrated in rural regions of the Western CONUS, as well as urban centers such as Los Angeles, San Francisco, Seattle and Vancouver, as captured in the annual prediction maps (Fig. S9 and Fig. S10).

### Increasing impact of wildland fire smoke on air quality and population exposure

**Figure 3:**
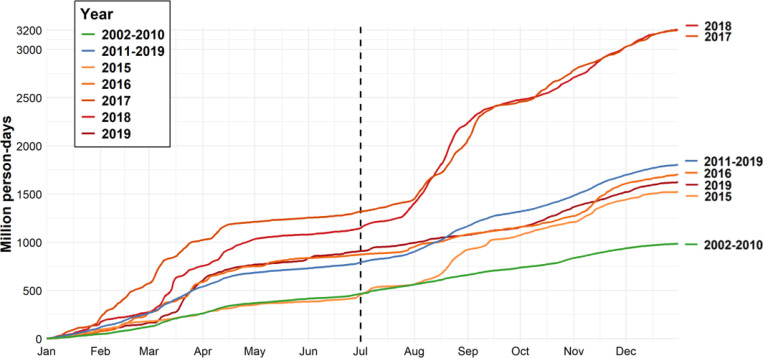
Cumulative person-days exposed to heavy fire smoke in the CONUS and SC (Unit: million person-day).

We evaluated the populations affected by heavy fire smoke in the CONUS and SC. A “heavy fire smoke grid-day” was defined as a grid-day when the total carbonaceous PM_2.5_ concentration exceeded 1 μg/m^3^ and the smoke carbonaceous PM_2.5_ constituted more than 50% of the total concentration. We compared the cumulative daily populations affected by heavy fire smoke during the periods 2002–2010, 2011–2019, and specifically the five years from 2015–2019 (Fig. 3). Our analysis revealed a clear increasing trend in population exposed over the years, corresponding to the broadening of wildfire smoke impacts. During 2002–2010, there was an average of 467 million person-days of exposure to heavy fire smoke prior to July 1st, while this number rose by 70% to 793 million person-days during 2011–2019. By year’s end, cumulative exposure increased from 986 million person-days in 2002–2010 to 1.8 billion person-days in 2011–2019, representing an 83% increase. To account for the influence of population growth on these trends, we also calculated the annual exposure days per capita. On average, individuals experienced 3.0 days of heavy smoke exposure per year in 2002–2010, which increased to 5.1 days per year in 2011–2019. The megafire years of 2017 and 2018 had particularly severe impacts, with cumulative exposure exceeding 3 billion person-days (Fig. 3), equivalent to 9.0 exposure days per person per year. Separate figures for cumulative person-days of exposure to heavy fire smoke in the CONUS and SC across years are provided in Fig. S11.

### Excess mortality due to smoke exposure

**Figure 4:**
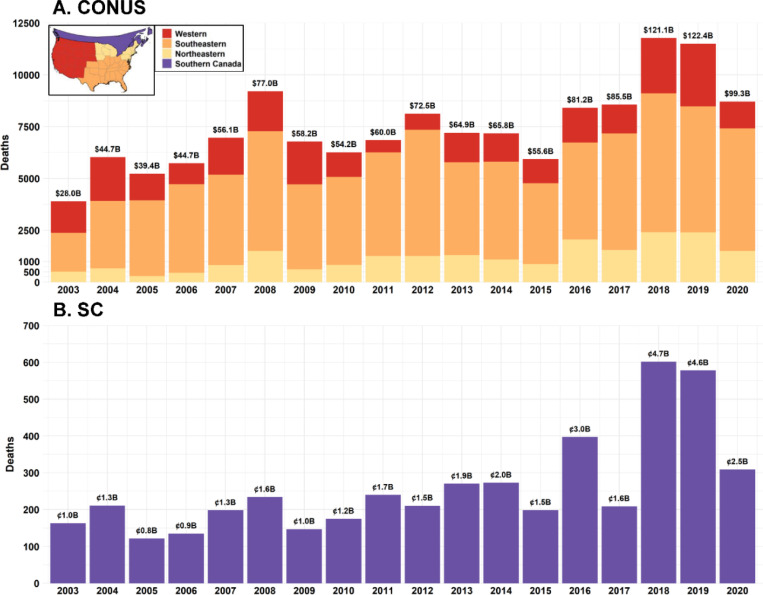
Annual total deaths attributable to fire smoke carbonaceous PM_2.5_ in the CONUS (subplot-A) and SC (subplot-B). **Note:** Climate regions in the CONUS were grouped into three major regions based on the U.S. National Prescribed Fire Use Survey Report and our analysis of smoke-impacted areas (see Fig. S5). These regions are defined as follows: the western region (i.e., climate regions: West, SouthWest, NorthWest, and West North Central), the southeastern region (i.e., climate regions: SouthEast, Central, and South), and the northeastern region (i.e., climate regions: NorthEast, East North Central). The annual total monetized damages for the CONUS and SC are labeled at the top of each column (units: billion USD for the CONUS and billion CAD for SC).

The annual non-accidental mortality rate and total deaths attributable to smoke carbonaceous PM_2.5_ from 2003 to 2020 are mapped at the county level in the CONUS and the census division (CD) level in SC (Fig. S12). Consistent with the spatial distribution of high smoke carbonaceous PM_2.5_ concentrations in the Western and Southeastern CONUS and Southwestern Canada, counties and CDs in these areas exhibited elevated annual mortality rates, exceeding 3 deaths per 100,000 people (Fig. S12A). The CONUS counties with higher annual death counts (> 3 deaths per year) were generally located in California, Oregon, Washington, Florida, Georgia, South Carolina, North Carolina, and Texas (Fig. S12B), areas characterized by high populations density, frequent smoke impact and elevated smoke carbonaceous PM_2.5_. On average, 7,462 non-accidental deaths per year in the CONUS were attributable to long-term exposure to fire smoke carbonaceous PM_2.5_ (Table S8), with the southeastern CONUS contributing 4,702 deaths per year (63.0%), and the western region contributing 1,568 deaths per year (21.0%). In SC, higher annual deaths were observed in urban areas of British Columbia, Ontario, and Quebec (Fig. S12B), with an average of 259 non-accidental deaths per year (Table S9). Annual total deaths in the southeastern region remained relatively stable over the study period (Fig. 4), whereas deaths in the western and northeastern regions, as well as in SC, fluctuated significantly in response to variations in wildfire intensity.

The average monetized damages associated with these mortality estimates were approximately 68.4 billion USD per year for the CONUS and 1.97 billion CAD per year for SC (Table S8 and Table S9). The southeastern region contributed 43.0 billion USD annually, and the western region contributed 14.2 billion USD. In 2018 and 2019, both mortality and monetized damages nearly doubled compared to the average levels, leading to monetized damages exceeding 120 billion USD for the CONUS and 4.6 billion CAD for SC (Fig. 4).

## Discussion

To the best of our knowledge, this is the first study to model full-coverage concentration of fire smoke-derived carbonaceous PM_2.5_ with high spatial and temporal resolution across both the CONUS and SC. We developed a three-level RASL framework to estimate both daily and long-term smoke carbonaceous PM_2.5_ from 2002 to 2019. Our analysis identified frequent smoke impact and elevated concentrations of smoke-derived carbonaceous PM_2.5_ in the Western and Southeastern CONUS, as well as in SC. Over the past decade, wildfire seasons have started earlier and intensified, resulting in increased population exposure to wildfire smoke, particularly during recent megafire years. We estimated that long-term exposure to smoke carbonaceous PM_2.5_ resulted in an average of 7,462 and 259 non-accidental deaths per year in the CONUS and SC, respectively, with the Southeastern CONUS contributing the most deaths.

The long-term concentration of PM_2.5_ mass from fire smoke in the CONUS and Canada has been examined in previous literature. Our findings on the spatial and temporal patterns of smoke carbonaceous PM_2.5_ are consistent with these prior studies. For example, Childs et al. estimated smoke PM_2.5_ over the CONUS at a 10 km resolution and observed increased smoke pollution and smoke-impacted days over the last decade, especially in the Western U.S. and in the years 2017 and 2018 (42). The FireWork model provided real-time forecasts of biomass burning PM_2.5_ across North America, showing that most wildfire events were concentrated in the Western CONUS, as well as in Western, Northern, and Central Canada (43). While previous studies have offered valuable insights into wildfire smoke PM_2.5_, our use of daily 1 km^2^ resolution and focus on carbonaceous PM_2.5_ provides a more precise accounting of fire smoke impacts and a more detailed understanding of its composition. Notably, our study revealed that the frequent smoke days and elevated concentration in the Southeastern CONUS are consistent over time due to the frequent use of prescribed burns, which has not been extensively discussed in prior wildfire smoke modeling studies or government datasets. For example, shortcomings were identified in the current prescribed fire permit databases given that some states in the Southeastern U.S. do not require prescribed burn permits and rely on voluntary reporting, such as Texas, Arkansas, and Missouri (39). Despite these limitations, our study successfully captured the elevated smoke days and smoke carbonaceous PM_2.5_ in these states.

Additionally, our results indicated a significant intensification of wildfires with higher concentrations of smoke carbonaceous PM_2.5_ across our study period, which has offset nearly one-third of the improvements in background air quality across the CONUS and SC, largely due to efforts such as the Clean Air Act (44). This intensifying trend in wildfire smoke stagnated or even reversed the declining trend of background concentrations in most regions. Our findings align with existing literature on smoke PM_2.5_ mass. Burke et al. reported that areas affected by wildfire smoke have doubled over the past two decades in the CONUS, and that wildfire smoke has influenced the average annual PM_2.5_ trend in nearly three-quarters of the states in the CONUS since 2016, accounting for 25% of the improvement in air quality (4, 45). However, few studies have investigated the wildfire smoke impact on PM_2.5_ concentration levels in Canada. Our results provide insights into wildfire smoke dynamics in Canada and the transboundary smoke effects. Nonetheless, additional research encompassing Alaska, Northern Canada, and Mexico is needed to achieve a more comprehensive understanding of the wildfire smoke activity across North America.

In addition to the increased smoke concentration, our findings indicate that exposure to wildfire smoke is becoming more common, implying more frequent wildfire smoke impact, wider smoke-impacted areas, and earlier and longer wildfire seasons. Consequently, the duration of exposure has lengthened, increasing from an average of 3.0 exposure days per year exposed to heavy fire smoke during 2002–2010 to 5.1 days per year during 2011–2019 for population across our study domain. Climate change plays a pivotal role in these shifts, as rising temperatures and prolonged droughts have shifted season regimes and created more favorable conditions for wildfires by drying out vegetation and extending the wildfire season (46–51). These conditions also facilitate megafires becoming more common, leading to extremely high concentrations of carbonaceous PM_2.5_. Our study exhibited significantly higher concentration levels and wider population exposure during megafire years such as 2017 and 2018, leading to increased health burden. Without significant mitigation efforts, future climate models predict an alarming increase in wildfire frequency and severity, which poses further risks to ecosystems, air quality, and public health (52, 53).

Prescribed fires are widely recognized as one of the most effective ways to prevent potential wildfires and sustain biodiversity in all regions beyond the Southeastern CONUS (54–56). Our study has revealed lower concentrations of smoke carbonaceous PM_2.5_ in the Southeastern CONUS during megafire years compared to the Western CONUS. However, our analysis also indicates that prescribed fires do not necessarily translate into better air quality and may lead to consistent smoke pollution with elevated carbonaceous PM_2.5_ concentration over time, causing adverse health effects from long-term exposure. In the Southeastern CONUS region, where prescribed fires are the primary sources of smoke, we estimated 4,702 attributable deaths per year, which is higher than the combined deaths in the Western CONUS (1,568 deaths per year) and Northeastern CONUS (1,192 deaths per year) regions. Several factors contribute to this outcome. First, unlike the Western U.S., where wildfires occur sporadically but at high intensity, the Southeastern CONUS experiences regular smoke pollution from frequent prescribed fires. Our results indicated that annual regional average concentrations of smoke carbonaceous PM_2.5_ in the Southeast CONUS are comparable to those in the Western CONUS (~ 0.4 μg/m^3^). While prescribed fires effectively reduce wildfires risks, they also result in frequent and localized smoke pollution in the Southeastern CONUS, where prescribed burns are conducted throughout the year (39). Second, prescribed fire smoke frequently affects densely populated urban and suburban areas in the Southeast CONUS, such as Atlanta and Charlotte, even though the fires are smaller and controlled (39). In contrast, wildfires in the Western CONUS typically occur in more remote, forested areas, such as the Cascades and Rocky Mountains. While wildfire smoke can travel long distances to urban centers like Los Angeles, San Francisco, and even the Northeastern CONUS, the primary impact is often concentrated in less densely populated areas. Third, residents of Southeastern states such as Georgia and Florida are more accustomed to prescribed fires (57). Engebretson et al. reported that Southern-state residents demonstrate significantly higher tolerance of potential health impacts from prescribed fires compared to those in Western states (58). While this acceptance reduces social barriers to the use of prescribed fires, it also lowers public vigilance regarding exposure to fire smoke pollutants. In contrast, the perception of wildfire risk in the Western U.S. has been heightened by the prevalence of megafires and media coverage, leading to a higher public awareness of the dangers posed by wildfire smoke and harm-reduction behaviors.

The average monetized damages associated with the attributable deaths in the Southeastern U.S. is 43.0 billion USD per year, and this cost for the CONUS has exceeded 120 billion USD in recent megafire years. In contrast, the U.S. allocated $1.73 billion to wildland fire management in 2024, with $214.5 million dedicated to fuels management (59). Considerable evidence in the scientific literature supports prescribed fire as a cheap and effective method for mitigating wildfire risk and reducing carbon emissions (60–62). However, most of these studies overlook the significant health impacts associated with smoke exposure from prescribed fire, which can be transported to nearby populated areas. When considering health-related costs, the cost-effectiveness of prescribed burns is called into question, as these fires can cause damage over 200 times greater than the budget. To better inform policy, it is crucial to develop a more accurate and comprehensive cost-benefit assessment for prescribed burns by incorporating health-related monetized damages from smoke exposure. Additionally, policies should prioritize minimizing human smoke exposure by improving monitoring networks of both PM_2.5_ mass and carbonaceous components and preparing communities for potential health impacts. Enhancing communication strategies to warn residents and provide resources, such as air quality alerts and protective equipment, should be an essential component of these policies. Beyond policy improvements, a more sophisticated prescribed fire management system is necessary — one that considers the conditions of each fire, including risk factors such as weather and proximity to populations, and the long-term benefits of prescribed burns in reducing the severity and frequency of wildfires (63). Achieving this balance between prescribed fire and public health is essential to ensure that prescribed burns remain a valuable tool for ecosystem health and wildfire prevention.

Our study has several implications. First, it provides a high-resolution fire smoke product with full coverage for the CONUS and SC, offering insights into the occurrence and distribution of fire smoke impacts, and quantitative estimates of background and smoke carbonaceous PM_2.5_ concentrations. The comprehensive spatial and temporal coverage of our predictions will enable future research on the health and environmental impacts of exposure to altered PM_2.5_ composition by fire smoke. Second, our findings highlight that wildland fires have intensified over the past decade, leading to an increase in deaths associated with long-term exposure to smoke carbonaceous PM_2.5_. Finally, by combining the prescribed fire permit databases with our study’s high-resolution smoke predictions, future efforts could better track prescribed fire activities in terms of locations, durations, sizes and transmissions. As climate change continues to challenge wildfire risk mitigation and biodiversity conservation, our study underscores the importance of incorporating potential health impacts in the cost-benefit analysis of prescribed fire policies and management tools.

Several limitations of our study should be noted. First, there is currently no research specifically investigating the mortality attributable to smoke carbonaceous PM_2.5_. As a result, our study relies on mortality risk estimates based on total smoke PM_2.5_. As carbonaceous PM_2.5_ is an important component influencing PM_2.5_ toxicity and a key contributor to all-cause mortality, using the carbonaceous fraction as a proxy for total smoke PM_2.5_ may lead to an underestimation of non-accidental deaths and monetized damages attributable to smoke carbonaceous PM_2.5_. Future research focusing on the mortality risk associated with specific components of smoke PM_2.5_ is needed to more accurately estimate the impact of carbonaceous PM_2.5_ from fire smoke. Second, the annual smoke-mortality relationships for both the CONUS and SC applied in our study may not be entirely applicable to the 2020 baseline mortality rate, which was impacted by the COVID pandemic.

In conclusion, our study highlights the growing impact of fire smoke carbonaceous PM_2.5_ and its adverse effects on public health across the CONUS and SC. With wildfires intensifying and becoming more frequent due to climate change, our findings underscore the urgent need for comprehensive prescribed fire management strategies that balance ecological benefits with the reduction of smoke-related health risks. This work provides a valuable foundation for future research and policymaking to address the dual challenges of wildfire prevention and public health protection in an increasingly fire-prone environment.

## Materials and Methods

### Study domain and Period

**Figure 5:**
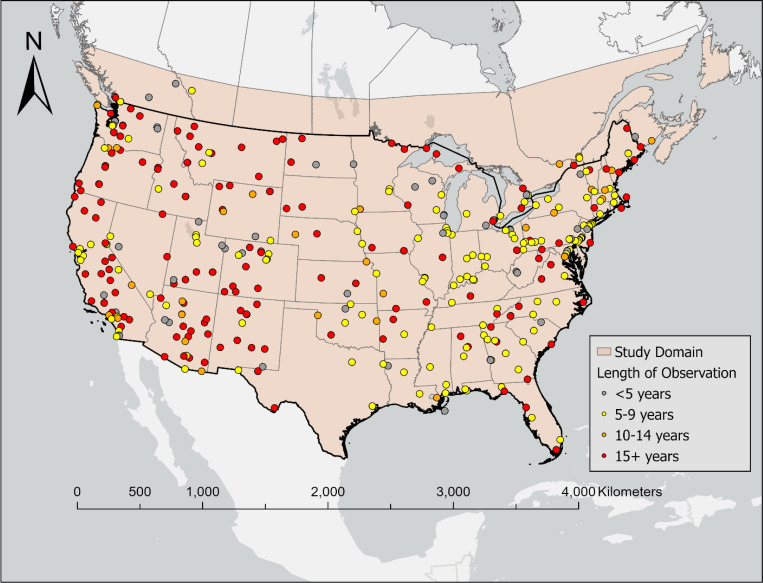
Study domain and ground-based monitoring networks of PM_2.5_ OC and EC.

Our study domain included the CONUS and SC (Fig. 5). The daily predictions were developed at a 1 km^2^ spatial resolution. In total, our modeling grid included 9,115,328 1 km^2^ grid cells for the CONUS and 2,620,640 1 km^2^ grid cells for SC, from 2002 to 2019.

#### Ground-based Carbonaceous PM Measurements

The ground-based measurements of carbonaceous PM_2.5_ (i.e., OC and EC) in the CONUS were obtained from a variety of sources, including the chemical speciation network (CSN) from Environmental Protection Agency (EPA), the National Park Service interagency monitoring of protected visual environments (IMPROVE) network, and the Southeastern Aerosol Research and Characterization Study (SEARCH) network (64, 65). For SC, ground-based observations were provided by the National Air Pollution Surveillance (NAPS) program (66). In total, 401 monitors operated during our study period, with most stations operating for more than 5 years (Fig. 5). Extremely high observations (> 99.98th percentile) of PM_2.5_ OC and EC were excluded from the model training to minimize the outliers (99.98th percentile of OC and EC: 34.75 μg/m^3^ and 7.40 μg/m^3^). PM_2.5_ OC and EC stations are often co-located and take samples synchronously, with similar number of stations and observations overtime (Table S10). Our final data includes approximately 448,000 daily measurements for OC or EC. Detailed summary statistics of ground-based measurements for training dataset are provided in Table S11 and Table S12.

### Chemical Transport Model

The state-of-the-art Community Multiscale Air Quality (CMAQ; www.epa.gov/cmaq) modeling system estimates atmospheric concentrations of numerous chemicals and aerosols, including ozone (and its precursors), PM_2.5_, and deposition of harmful chemical species (67). CMAQ is developed and maintained by the US Environmental Protection Agency (EPA), and it has been widely used for assessing air pollution, including evaluating policies and estimating impacts on human health (68–70). In this study, we used CMAQ version 5.3.2 and the datasets from the EPA’s air QUAlity TimE Series (EQUATES; www.epa.gov/cmaq/equates) project (71).

In this study, we used two sets of daily simulations at 12 km^2^ spatial resolution for the period of 2002–2019: (1) the baseline CMAQ simulation results from EQUATES, which includes all emission sources, and (2) a sensitivity simulation conducted using the same configurations (Table S13) and inputs as in the EQUATES project, except by not including fire emissions. Hereafter, these two sets of simulations are referred to as “Smoke-on” and “Smoke-off”, respectively.

### Classification of Smoke Impacted Areas

To better account for the specific contribution of fire smoke to PM_2.5_ OC and EC concentrations, we utilized CMAQ simulations run under the different settings. The grid cells in the study domain were classified into two daily smoke scenarios: a background scenario without fire smoke impact (“Smoke-off”) and a fire smoke-impacted scenario (“Smoke-on”). The Smoke-on CMAQ model simulations were used in modeling carbonaceous PM_2.5_ under Smoke-on scenarios, while the Smoke-off CMAQ model simulations were used for Smoke-off scenarios. Detailed classification methods are provided in the Supplementary Document S1.

### Auxiliary Predictors

To enhance model performance and predictive accuracy, we incorporated a wide range of auxiliary predictors in model development. These predictors included satellite-retrieved aerosol data products, cloud coverage, and smoke plume information, gridded meteorological factors, vegetation coverage, biogenic emissions, population, land cover, road density, topographic data, human footprint, and coordinate and time trend characteristics. These variables have been found to be important predictors in prior studies (36, 42, 72, 73).

All predictors at different spatial resolutions were integrated into the 1 km^2^ grid cells obtained from the Multi-Angle Implementation of Atmospheric Correction (MAIAC) dataset, which served as the grid template for the MAIAC aerosol optical depth (AOD) measurements (74). Daily ground-based measurements of PM_2.5_ OC and EC were assigned to their collocated grid cells. Detailed descriptions of the data sources and process steps are provided in the Supplementary Document S2.

### Modeling framework

**Figure 6:**
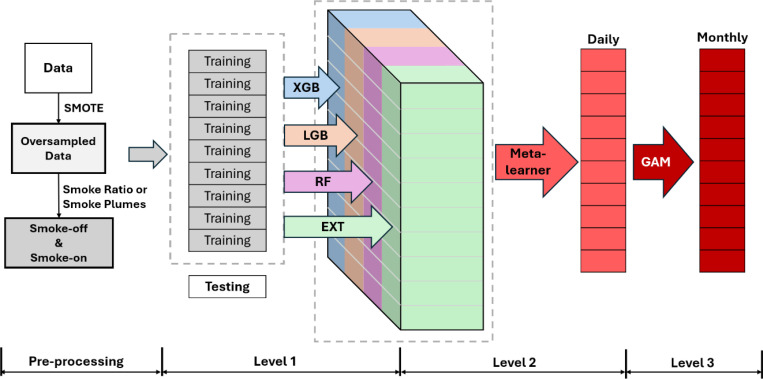
Modeling framework of the three-level residual adjusted super learner (RASL).

After aggregating the raw measurements for OC and EC, we applied the Synthetic Minority Oversampling Technique (SMOTE) to oversample the underrepresented high-concentration measurements (75). The enriched training datasets were then classified into Smoke-off and Smoke-on scenarios. For both PM_2.5_ OC and EC, and both smoke scenarios (Smoke-off and Smoke-on), we employed the proposed Residual Adjusted Super Learner (RASL) framework (Fig. 6). This framework operates with a three-level structure based on the super learner method, in which the cross-validated predictions from four base models (first level) were incorporated using a meta-learner algorithm (second level) to generate fused predictions of carbonaceous PM_2.5_ (76). Finally, generalized additive models (GAMs; third level) were used to adjust the spatiotemporal residuals of monthly concentrations at each 1km grid cell from 2002 to 2019 across the study domain. Details of the modeling framework are provided in the Supplementary Document S3.

### Model Performance Evaluation

We conducted a three-stage cross-validation (CV) to evaluate the model performance at each level of the RASL framework. Three types of CV were employed: 10-fold random CV, 10-fold clustered spatial CV, and leave-one-year-out temporal CV. The clustered spatial CV better tests the model’s predictive ability when a large group of monitoring networks is missing instead of a single monitor (77, 78). Prediction accuracy was evaluated using three metrics: the coefficient of determination (R^2^), root-mean-square error (RMSE), and slope. To further evaluate the model at different time scales, we averaged daily estimations into monthly and annual values, allowing us to capture both long-term trends and short-term fluctuations—important for long-term cohort studies and short-term analyses. Details of the CV experiments are provided in the Supplementary Document S4.

### Calculation of mortality burden and monetized damage

We employed different methods in the CONUS and SC to calculate the non-accidental mortality and monetized damages attributable to fire smoke carbonaceous PM_2.5_. For the CONUS, Ma et al provided the monthly non-accidental mortality rates for different smoke PM_2.5_ concentration bins in the CONUS (20), which we then multiplied by 12 months to estimate the annual mortality rate (Table S14). The annual mortality rate, along with each year’s smoke concentrations and population data, was used to estimate the following year’s deaths. To assess the monetized damages associated with these mortality estimates, we employed the Value of Statistical Life (VSL), as provided by the U.S. Department of Health and Human Services. VSLs reflect the monetary value that individuals are willing to pay to reduce the risk of death, thereby providing an economic perspective on mortality burden. We based our estimates on the 2013 VSL value and then adjusted it annually from 2003 to 2020 in accordance with HHS guidelines (Table S15), to account for inflation and changes in real income for the specific dollar year (79). The year-specific VSL values were multiplied by the estimated mortality attributable to fire smoke carbonaceous PM_2.5_ exposure in that year to provide an estimate of annual monetized damages.

For SC, we applied the Air Quality Benefits Assessment Tool (AQBAT) developed by Health Canada, which is designed to estimate the human health impacts and economic valuation of changes in Canada’s ambient air quality (80). AQBAT provided the concentration response functions between chronic exposure to fire smoke PM_2.5_ and mortality and, which enabled us to estimate the number of attributable mortality and related monetized damages across Canada (Table S9).

## Supplementary Material

Supplement 1
