# Supplementary material for: Fire Smoke Elevated the Carbonaceous PM2.5 Concentration and Mortality Burden in the Contiguous U.S. and Southern Canada
Source: Res Sq. 2024 Nov 21:rs.3.rs-5478994. Preprint. [Version 1] doi: 10.21203/rs.3.rs-5478994/v1 (PMC11601856; doi:10.21203/rs.3.rs-5478994/v1)
Supplement: Supplement 1 [file NIHPPRS5478994V1-supplememt-1.pdf]

Supplementary Files

This is a list of supplementary files associated with this preprint. Click to download.

- SmokeCarbonPM25SupplementaryRS.docx
